# The Simcyp Population Based Simulator: Architecture, Implementation, and Quality Assurance

**DOI:** 10.1186/2193-9616-1-9

**Published:** 2013-06-03

**Authors:** Masoud Jamei, Steve Marciniak, Duncan Edwards, Kris Wragg, Kairui Feng, Adrian Barnett, Amin Rostami-Hodjegan

**Affiliations:** Simcyp Limited (a Certara Company), Blades Enterprise Centre, John Street, Sheffield, S2 4SU UK; Centre of Applied Pharmacokinetic Research, the School of Pharmacy and Pharmaceutical Sciences, the University of Manchester, Manchester, UK

**Keywords:** ADME, Pharmacokinetics, Pharmacodynamics, Physiologically-based pharmacokinetic, Simcyp, Model based drug development

## Abstract

Developing a user-friendly platform that can handle a vast number of complex physiologically based pharmacokinetic and pharmacodynamic (PBPK/PD) models both for conventional small molecules and larger biologic drugs is a substantial challenge. Over the last decade the Simcyp Population Based Simulator has gained popularity in major pharmaceutical companies (70% of top 40 - in term of R&D spending). Under the Simcyp Consortium guidance, it has evolved from a simple drug-drug interaction tool to a sophisticated and comprehensive Model Based Drug Development (MBDD) platform that covers a broad range of applications spanning from early drug discovery to late drug development. This article provides an update on the latest architectural and implementation developments within the Simulator. Interconnection between peripheral modules, the dynamic model building process and compound and population data handling are all described. The Simcyp Data Management (SDM) system, which contains the system and drug databases, can help with implementing quality standards by seamless integration and tracking of any changes. This also helps with internal approval procedures, validation and auto-testing of the new implemented models and algorithms, an area of high interest to regulatory bodies.

## Background

The relative complexity of differential equations involved in physiologically based pharmacokinetic and pharmacodynamic (PBPK/PD) models, together with the need for comprehensive drug and system data, has previously limited their use to a small group of modelling scientists; however such models have been around since the 1930s. (Teorell 1937). Recently, applications and acceptance of PBPK models joined with *In Vitro* – *In Vivo* Extrapolation (IVIVE) of Absorption, Distribution, Metabolism and Excretion (ADME) in drug development and regulatory assessment have significantly increased (Zhao et al. 2011 Rostami-Hodjegan 2012). This trend can be attributed to many factors, including, the availability of user-friendly software and an increase in computing power, which have facilitated the use of PBPK modelling (Rowland et al. 2011). Bouzom and co-workers reviewed the features and limitations of both off-the-shelf and the traditional user customisable software packages which are frequently used for building and applying PBPK models (Bouzom et al. 2012). The Simcyp Population Based Simulator is a commercially available package used for Model Based Drug Development (MBDD). An overview of the framework and organisation of the Simulator and how it combines different categories of information was previously described (Jamei et al. 2009a). Each year more than 40 man-years of effort go into updating and refining the Simulator; hence it is necessary to provide an update on the latest architectural and implementation developments. Interconnection between peripheral modules, the dynamic model building process and compound and population data handling are described. Also, the Simcyp Data Management (SDM) system, which contains the system and drug databases and facilitates implementation of quality assurance procedures, is explained. This article also provides details of the regression autotesting procedures implemented to ensure the consistency and integrity of new models and their interaction with already implemented modules.

### Chronology of modules

**Figure 1:**
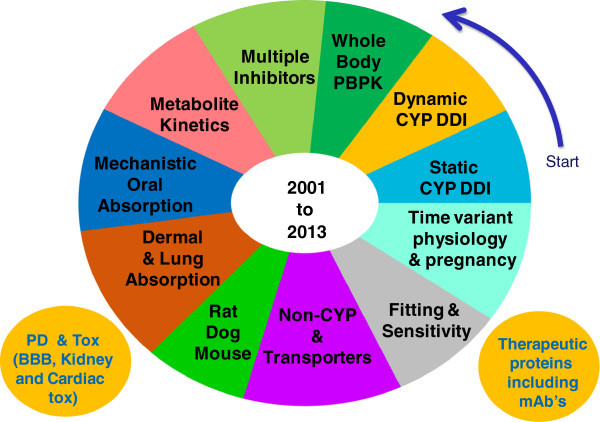
**The chronology of expansion of the Simulator features from 2001–2013 under the Simcyp Consortium guidance.** The development started with static metabolic drug-drug interaction calculations then dynamic drug-drug interaction models followed by whole body PBPK and so on.

Since its inception the Simcyp Simulator has been developed in conjunction with a Consortium of major pharmaceutical companies, who share pre-competitive information and provide guidance on the addition of further capabilities and features. The usage and functionality of the Simulator is further enhanced by collaborations with drug regulatory bodies and academic centres of excellence worldwide. Every year a new version of the Simulator with new features is released. A rough chronological order of the development of models and databases within the Simulator is shown in Figure 1. The Simulator development started with simple static drug-drug interaction calculations (“Static CYP DDI” section of Figure 1). This was expanded to dynamic models and the minimal PBPK model that was subsequently expanded to full PBPK models. The recent developments include handling of therapeutic proteins, the ability to add custom PD scripts and to model time-variant physiology in paediatric population as the subjects grow and in pregnant women over the duration of pregnancy.

Various architectural and procedural modifications and enhancements introduced to maintain performance and minimise simulation time are explained in the following sections. The general workflow, quality assurance procedures and module testing methodologies are also described.

## Methods

Each year a new version of the Simulator is developed and released. This requires a rapid application development environment that allows many iterations of code within a development cycle to present and implement novel modules in the most expedient manner. This naturally dictates a specific workflow and procedures for dealing with model development/implementation and data generation/maintenance. The development workflow, data structure and integrity check, dynamic model building and testing procedures are explained in this section.

### Development workflow

#### Agile and scrum

From the early days of development in 2002, an approach to software development has been used which maps quite closely to methodologies known as Agile (Martin 2011 Larman and Basili 2003). For Simcyp, this approach usually involves a group of scientists, who are responsible for creating a design or implementation document for the new feature, along with at least one developer. Each project team has a lead person who heads the team and coordinates their efforts to define and deliver the project deliverables within the given timeframe. Each project also has a supervisor who provides advice and guidance whenever needed and also is responsible for evaluating scientific and technical aspects of the deliverables and approve the implementation documents before they are passed onto developers for implementation.

Although the implementation document contains the scientific requirements of the new feature it is not possible to capture the nuances of interfacing with multiple other concurrent projects nor can it capture interfacing with an ever evolving computational core. As such the project development is carried out in small sections with iterations to modify and embed the new feature. In order to accomplish this smoothly a Scrum approach (Sims and Johnson 2012) is taken where regular updates aim to ensure that developers working on different projects are aware of what other projects and developers are both doing and planning to do in the short term. This highly communicative approach can remove a large amount of ambiguity and ‘surprise’ during the development cycles.

#### Microsoft visual studio and team foundation server (TFS)

The Simcyp Simulator is currently developed using Microsoft Visual Studio 2010. Development is split into two principal areas, the addition of new functionality and the updating of existing features. As a result, multiple developers may be working on similar areas of code that could in practice lead to significant development collisions. To counteract this and to implement rigid quality control procedures Microsoft Team Foundation Server (TFS) is used as the central code repository but also as the principal marshalling tool for code development (Guckenheimer and Loje 2012).

Addition of code to the Simulator is strictly controlled. Firstly, no code can be added to the Simulator without being linked to a Task (see next section). The Task is a unit of work defined within TFS and relates to either a Project that is under way or a bug fix or an enhancement. In this way there is traceability between documentation and lines of code. Secondly the code being checked in must ‘merge’ with the current code that is in the repository. It is highly probable that another developer may have been working on the same files at the same time. This could lead to collisions where one developer modifies or overwrites another’s code changes. TFS prevents this through its intelligent Merge tools and by calling on the developer to make executive decisions where merging becomes ambiguous. As each code section is added a build is carried out to verify the integrity of the added code and ensure the overall code is never ‘broken’.

#### Projects and tasks

With the increasing use of Simcyp in regulatory submissions, full code control and audit trails need to be rigorously imposed. To achieve this aim a policy of no code development without accompanying approved documentation has been introduced (see next section). In order to marshal the documentation, TFS is used to provide a direct link from the project based documentation or the ‘bugs database’ through to the code that is developed.

In the first instance, the project team develops an implementation document which is approved by two supervisors (the project supervisor and another supervisor who only checks/approves the final deliverables) before it is passed onto developers.

This document is used to generate an overarching Task within TFS. This Task itself can have Subtasks dependent on the complexity of the project, for example Graphical User Interfaces (GUI) development, algorithms subtasks, outputs etc. All code that is then generated by the development team has to be linked to the Task or Subtask thus providing a path between lines of code and the implementation document.

Following on from first stage implementation there may be a need to fix bugs and add enhancements. TFS has the facility to report a bug or request additional features that is accessible by all developers as well as the scientists who are involved in the design and testing of the platform. This allows for the raising and tracking of reported/fixed bugs.

### Data structure and integrity check

Inherent in the design of the Simcyp Simulator is a separation of data within discrete files from a relatively benign GUI that operates on those files and an Engine that uses file-based inputs as its ‘menu of operations’. Apart from being in line with systems pharmacology concepts (in term of separation of systems, drug and trial design data), this has a number of significant benefits: notably the ability to share discrete collections of data more easily within data libraries, to develop fast throughput systems minimising user interaction and perhaps the most important, an ability to develop an automated testing strategy which both reduces testing time and ensures a greater integrity of the testing process.

### Data files structure

The design of the Simcyp Simulator has been based around the portability of the underlying data. In other words all compound and population information can be fed into Simcyp as external data files. There are two principal classes of data that are necessary to run any simulation, namely compound data and population data. These data classes respectively correspond to properties of (or values dependent on) a particular compound, and to data describing the demographic, physiology, genotypic and phenotypic characteristics of a specific ethnic or disease population with relevant inter-individual variability distributions.

These two types of data are conveniently stored within XML-based files that can be viewed and accessed via the Simcyp GUI as well as other tools such as Microsoft Internet Explorer. The schema of these files has been designed internally to allow forwards compatibility of files over time. In other words the schema is fixed with each release version and new parameters that are added are done so without disrupting what already exists. This allows files created with a version of the Simulator today to be used with later versions when they come out where any possible missing values are automatically replaced with default values. All files contain a degree of meta-data showing varying information including the Simulator version used to create the file. In order to ensure users are aware of changes a warning message is displayed indicating some data have been added/updated and they should consult with the help file.

### Workspaces

The compound and population data files have limitations in that neither captures the simulation trial conditions nor the user optimisation options utilised on the user screens. To cater for this a third type of file structure known as a workspace has been developed. Again the workspace file is XML-based; however this time it acts as a container for one or more compound files, one or more population files as well as all trial information and user defined settings. Because of its all-encompassing nature it is used as a snapshot of the running condition of any simulation. In other words, to reproduce any simulation exactly, all that is needed is a copy of the workspace taken at the time the simulation was run.

As a Workspace contains full details of the simulation, it is possible to use the workspace file alone to drive a simulation. In this manner the screens can become detached from the main engine enabling much greater flexibility in designing and updating the interface. Another benefit of defining workspaces is the ability to run a simulation in an automated/fast throughput mode.

### Reporting simulation results

After running simulations Microsoft Excel (Microsoft Corp., Redmond (WA), USA), is used as the output medium to present the results. Excel was selected because the majority of computers in the workplace would have Microsoft Office software installed and additionally Excel contains many powerful analysis tools that could be used to analyse the output data once in Excel.

The reporting process is implemented through the Excel Automation interface which is based on the Office Object Model. The Simcyp platform uses this technology to create or connect to an Excel application Component Object Model (COM) object, to manipulate and add worksheets as required. Each worksheet is a bespoke output based on the simulation input selections: each cell is effectively created individually with the selection of font (including size and weight), colour (both foreground and background), alignment of text within the cell, number format (based on the users’ machine selection) as well as many other specifications.

After the data have been rendered, Excel charts are added if applicable. These may be concentration-time profiles or, for example, pie charts of enzyme contribution which are created based on the data on the worksheet and formatted individually based on user selections such as number format and also the colour ‘skin’ chosen before outputting the data.

The cost of using the Automation interface is speed: sending large amounts of data across process boundaries is a great bottleneck. This can be improved using alternative methods such as creating the Excel file directly which means the whole process will be a simple file input/output process and hence much quicker. Unfortunately, the development time required for this is huge and the entire process would need a great deal of testing as this would require writing the code from scratch. Currently, an option is provided to write a subset of data to a CSV file. This is extremely quick and the data can subsequently be imported by most packages (including Excel). The down-side of CSV transfers is that no formatting or graphs can be included.

Other output options are the use of a relational database. This is currently used by the Simcyp Batch Processor but could be expanded to include standard Simcyp outputs. This would be a powerful tool to put the Simulator as part of a company workflow as it could then write directly into a corporate database. The downside of this is similar to the CSV option: all formatting and visualisation would have to be done by the user.

### Insertion of workspaces into excel

There is frequently a desire to reproduce a complete simulation from the results of an earlier simulation whether to verify original findings or to continue running similar simulations. In order to facilitate this, the Simcyp Simulator can embed a copy of the workspace into the Excel file while creating the Excel outputs. This is a two phase operation with the first phase being the creation of a workspace ‘on the fly’ after a simulation has taken place but before any output to the Excel file. The second phase is the embedding and subsequent encryption of the workspace within the Excel file. The embedded workspace can only be accessed by the Simulator at a later stage; therefore it is not possible to manipulate or change the workspace data, so it can be used as a quality control measure. Although the embedding of a workspace is desirable both in terms of internal traceability (input and output data fully interlocked) and when submitting results to authorities, there is a small overhead involved both in terms of reporting time and file size.

### Simcyp Data Management (SDM) system

Simcyp Ltd often release a number of new and revised Compound and Population files, typically on an annual basis. These files are the culmination of a large amount of meta-analysis and changes may include new parameters for previous files when new features are incorporated. This can result in variation between file contents from version to version.

There are two quality issues that surround the files themselves. The first is the curation of data. Both the Compound files and the Population files can each have in excess of 1000 data items and generation of each of these files is usually the outcome of a meta-analysis of multiple sources. In order to impose a level of control over this huge pool of data and to allow intelligent querying, a tool known as the Simcyp Data Management (SDM) system has been developed. This tool takes in the numbers that will go into the Simulator from scientists via whatever mechanism they exist in at the time and passes the data through a series of approval procedures before allocation to particular files. This happens for all values within a file, ensuring that a full history of the source of each number is maintained. As the system is database driven, it also enables searches to be carried out across files and versions to allow cataloguing of changes to take place rapidly. As an end point the SDM generates the compound and population files themselves that are then bundled with the Simcyp Simulator. This removes possibility of human error in the process and ensures data integrity.

These files are stored in an xml format. When new values are added to the files i.e. when new features are added, these new values are appended with no change to the existing structure. A big benefit of this feature is to enable version control while allowing a new release version to read an older version’s xml file.

### Model structure and testing

Simcyp PBPK models are built using ordinal differential equations (ODE). The models’ differential equations are handled in a module called ‘Simpak’ which is an independent and scalable environment to accommodate the continuing evolution of the models and algorithms contained within the Simulator (Jamei et al. 2009a). One of the main objectives in the Simulator’s design is to take away the model building burden from users. Therefore, users only select their desired models using the Simulator GUI and these selections are dynamically translated to the relevant models in ‘Simpak’. To allow more flexibility and scalability ‘Simpak’ is highly modularised to facilitate the combination of many different components. Currently, the Simulator handles over 1000 PK model combinations, involving:

-  Single (small and large molecules) or multiple (up to 4) chemical moieties,
-  Different absorption models, namely one-compartment, enhanced Compartmental Absorption and Transit (CAT), and Advanced Dissolution, Absorption and Metabolism absorption (ADAM) models,
-  Different distribution models such as minimal and full PBPK models with different perfusion- and permeability-limited models, including multi-compartment liver, kidney, blood–brain-barrier models and an additional multi-compartment user defined organ/tissue,
-  Modelling of up to 3 metabolites.

As an example, if the ADAM and full PBPK models are used while the gut, liver, brain and kidney transporters are incorporated for the substrate and main inhibitor around 500 differential equations are automatically assembled to run the simulation.

The ever increasing number of models and the exponential rise of possible combinations have made model checking/testing a challenging task; therefore a range of solutions for model testing has been deployed which are explained below.

### Regression testing

Reproducibility of simulation result is a key requirement when users install different versions of the Simulator and plays an equally important role during the development lifecycle of the Simulator. As the complexity of the Simulator has increased automated tools have had to be developed to allow a large proportion of the testing to be done automatically. The key players in this regime are the Simcyp Autotest package coupled with an in-house Excel Comparison tool; which compares the same worksheets in two given Excel workbooks; that enables a full regression testing (comparing the results from a new build against historical results) procedure to take place directly and automatically after any development build of the Simulator. For this purpose a repository of workspaces that is ever increasing, which cover a wide range of GUI options, is established and used during regression testing.

### Autotesting

The Simcyp Autotest package allows the automated running of multiple workspaces with pre-selection of relevant outputs. In order to make use of this as part of a regression testing, two areas need to be addressed. Firstly, any new build of the Simulator needs to generate similar results to those generated in a previous build for an existing feature in similar scenarios. Secondly, testing needs to be done to ensure any new feature needs to generate defined outputs without interfering with existing features in any unpredictable/unexpected manner.

To check this, existing results generated by running defined Workspaces on early versions/builds need to be checked against results from the current released build of the Simulator to ensure that no unexpected discrepancies (i.e. where the underlying algorithms and/or data have not been intentionally altered) occured. Additionally new features need to be run via newly defined Workspaces to ensure that initially a benchmark and then subsequently regression testing against that benchmark can take place. In practice, project teams initially manually verify the results generated by new modules often against previously developed prototypes in other environments such as the MATLAB (The Mathworks Inc., Natick (MA), USA) or R (1) packages. Further, project teams are asked to prepare a range of workspaces to cover required features. These workspaces will be repeatedly run for each successive build and compared to the previous run. The Autotest package was initially developed as Python Scripts but has since been recoded in C# to add extra layers of functionality. The exposed features within the Simulator Engine however allow any scripting engine to be used.

Over time, new literature data leads to the revision and updating of physiological and compound parameters used within the Simulator. When this happens, the workspace files are updated with the latest values. Consequently the corresponding benchmark test results are also modified for future regression testing.

The Autotest package provides two options for determining the outputs. The first and default method is to use the Excel outputs that the user defined at the time that the Workspace was saved. In this case the workspace serves as a complete input and output unit. The second method is to define the required outputs via command line inputs within the script. Although effective the latter approach can be time consuming given the large number of different sheets that can be generated.

**Figure 2:**
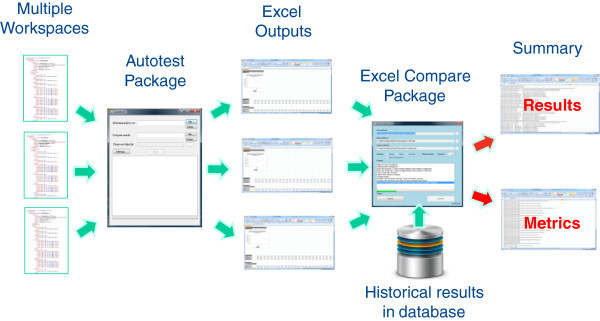
**The overall autotesting process which starts from running the repository of workspaces to the generation of summary reports.**

A final by-product of the Autotest system is metrics. A wide range of metrics are gathered for each simulation and can be compared against previous Autotest runs in order to ensure that either programmatic issues (memory usage, simulation run time, etc.) and/or algorithmic issues (stiff differential equations) have not crept into the design and had any adverse effect on time and/or memory usage. The overall autotesting process is shown in Figure 2.

### Excel comparison tool

An issue that occurred when running Autotest was the huge number of Excel files that are potentially generated as a result of running multiple workspaces across many projects. Since checking these files manually was very tedious and time consuming, a new in-house package was developed to compare one set of Excel files (the current run) against an earlier set. Built into this utility was a way of setting a threshold so that discrepancies could be reported on a master report sheet with colour coding added to the Excel files themselves to ‘mark’ the discrepancies. In this way a user can allow discrepancies of say 1% to exist between values to cater for potential rounding errors in Excel yet still report significant discrepancies. Similar checking is carried out on the metrics files to verify time or memory differences are not occurring.

### Code coverage testing as part of regression testing

Given the high number of model combinations and available options, there were cases where the workspaces did not cover specific areas. Hence, the question was how to make sure all areas of the code are sufficiently covered for testing. To address this issue and assess which parts of the code were activated whilst running a specific workspace, a code coverage tool called the Bullseye Coverage package (Bullseye Testing Technology, Washington, USA) is used. It determines which paths through the code within the new module are called and tested and which remain to be. Therefore, this additional checking assists with identifying any shortcomings in the test workspaces and allows an iterative approach to generating a good quality set of test workspaces that cover as many options as practical.

### Computer virus checking

The Simulator is usually used in an environment (pharmaceutical companies and regulatory agencies) where it is essential to be free from any computer viruses or malware and additionally for the software not to send any sensitive information outside of the company. Therefore, the Simulator (after passing all internal testing and checking and when it is ready to be released to clients) is sent out to the National Computing Centre (NCC). The NCC is an independent membership organisation for IT professionals and is the single largest and most diverse corporate membership body in the UK IT sector. The centre runs a variety of tests on all Simulators (for human and animal species) to verify they are free from any computer viruses or malicious codes and do not broadcast anything outside of the computer. The NCC then issues a certificate and endorses that specific release of the software after which the installer is released to users.

## Peripheral modules

During the course of Simulator development many additional (peripheral) modules were added to extend its features and functionality. To retain the modularity of the platform and facilitate its maintenance these modules were developed independently whenever possible and called in to perform specific tasks when needed. One of the very first peripheral modules was the Batch Processor that allows running many simulations without user intervention (Jamei et al. 2009a). In the current article three major peripheral modules added since Simcyp Version 9, namely the Automated Sensitivity Analysis (ASA) tool, Parameter Estimation (PE) modules and the Pharmacodynamics (PD) module, are described. Furthermore, an update is provided on the differential equation solver.

### Automated sensitivity analysis tool

**Figure 3:**
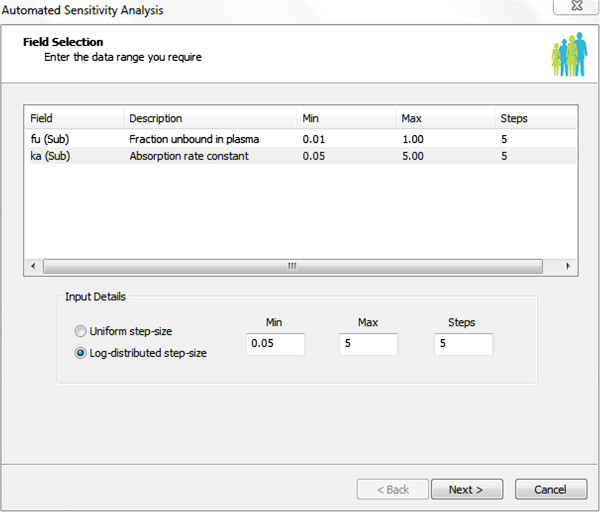
**A screen shot of the automated sensitivity analysis tool in Simcyp Version 12 Release 2; an example for assessing the impact of fraction unbound in plasma and the absorption rate constant on specific outputs where the minimum and maximum values, the steps and the step-size distributions are defined.**

On occasion there is uncertainty in the true value of an input parameter. This may be due to some particular parameter being unavailable for the drug of interest or because for that particular compound the *in vitro* data is unreliable. In these cases it is useful to check the impact that the input parameter has on the simulation outcome. This can be achieved using the automated sensitivity analysis (ASA) tool. This is a local sensitivity tool which scans the selected parameters within a given range and reports the selected endpoints for a population representative subject. ASA can be used to assess the impact of changing specific parameters (maximum of two at a time) on a range of PK/PD parameters or concentration-time profiles. For investigating more than two parameters the Batch processor (Jamei et al. 2009a) can be used instead. Identifying whether an input parameter has a significant impact on the outcome of a simulation is highly valuable as it assists with making decision on what *in vitro* assays should be done at what stages and how much resource should be invested in obtaining a particular parameter for a particular compound. ASA can be performed on virtually any parameter displayed on the Simulator interface. After a parameter is selected the ASA interface tool is called within this interface the user can define the parameter ranges and the number of steps the range should be divided into, as shown below (Figure 3).

The user then selects the endpoint parameters to be shown as Excel output, many of the PK/PD parameters and profiles are available for selection when ASA is used. Within the Excel outputs the selected endpoint values for any of the selected input values along with the sensitivity index, which is the ratio of change in endpoint to the change in input, and elasticity index, which measures the relative change in endpoint for a relative change in input, are provided. Generally, sensitivity analysis is recommended prior to fitting a model to the observed data as these help to decide which parameters should be or can be fitted.

### Parameter estimation (PE)

Another recently introduced feature of the Simulator is the ability to simultaneously estimate up to 10 parameters to match clinical observations using the Parameter Estimation (PE) module (Zhao et al. 2012). One of the biggest advantages of this module is that a user does not need to write any models and their relevant differential equations as all of these are automatically performed. This module allows non-modellers quickly and efficiently to fit very sophisticated models to clinical observations. Historically, model fitting has been an area of expertise that only a limited number of modellers could handle and this limited the wide use of modelling and simulation in the pharmaceutical industry. Introduction of modules such as PE has enabled a larger group of scientists to apply model-based drug development.

Since many PK covariates are already built into the Simulator (Jamei et al. 2009b), the covariate selection, which can be a challenging step in typical Nonlinear Mixed Effect (NLME) modelling, becomes a less challenging task. This is a major advantage of parameter estimation using models that have been developed in a systems pharmacology context.

To allow entering the observed clinical data, PE templates are designed and implemented as add-ins within Excel. The PE templates are structured in a manner that facilitates entering clinical data which are already saved in NONMEM (GloboMax, ICON Development Solutions, USA) software format. Within PE templates, two sets of data checking are carried out: first, while the data are being entered and second, right before saving the file to check the consistency of overall entered data. The location and reason of any errors are reported to users so they can quickly ratify them. At the end the data are saved in XML format.

The available on screen parameters are dynamically changed depending on the selected models and users can easily select parameters to be estimated from the screen which makes the fitting process intuitive. After selecting the parameters to be estimated invoking the PE module brings up an interface from which the optimisation methods, error models, ending criteria and the parameters' initial values and ranges can be entered. The PE can fit models using both rich and independent individual data and sparse population data as it is done in typical PoPPK data analysis.

**Figure 4:**
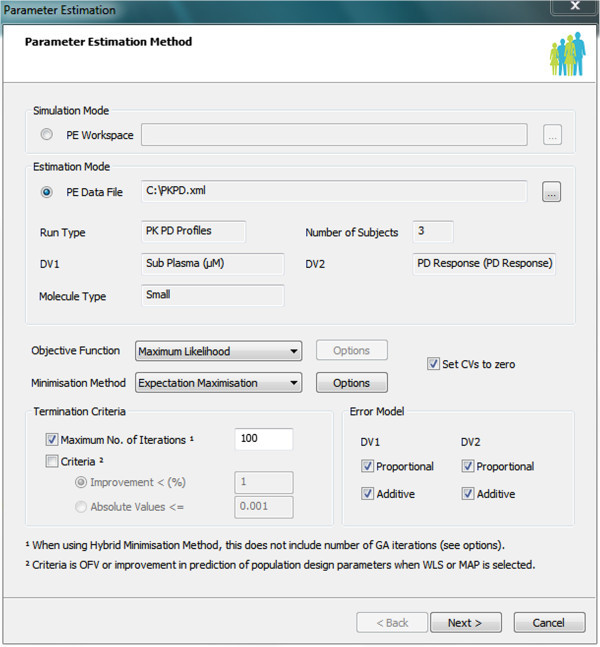
**A screen shot of the Parameter Estimation (PE) module that allows either of simulation or estimation modes.** The observed clinical data are loaded in XML format and in the shown case the data include both plasma concentration and a PD response profile for simultaneous fitting of PK and PD dependent variables.

A range of least square objective functions with different weighting methods can be selected for fitting. These objective functions can be fitted using classical Nelder-Mead (Nelder and Mead 1965) or Hooke-Jeeves (Hooke and Jeeves 1961) methods or more modern methods such as Genetic Algorithms (Goldberg 1989). For NLME fitting the Expectation-Maximisation (EM) (Dempster et al. 1977) method is used to solve either of the Maximum Likelihood or Maximum A Posterior problem. A screen shot of the main interface of the PE module is shown in Figure 4.

### The PD (pharmacodynamic) framework

Since Version 11 Simcyp has provided a collection of PD models for simulation and parameter estimation to complement PK/ADME modelling facilities. The initial emphasis in development of this PD module has been to capture some well-known semi-empirical models within building blocks (*PD Response Units*) which can readily be linked together by generalized transduction to represent more complex responses. This early framework envisaged connecting simulated compounds (PK substrate/inhibitor/metabolite) to one or more responses via a directed acyclic graph (DAG) of linked units. Complexities of PD to PK feedback and other systems biology motifs were to follow on a slower timescale. Similarly, mechanistic details of receptor-based *in vitro* to *in vivo* extrapolation (PD-IVIVE) were to be grafted on more gradually, as the science developed, within this framework.

**Figure 5:**
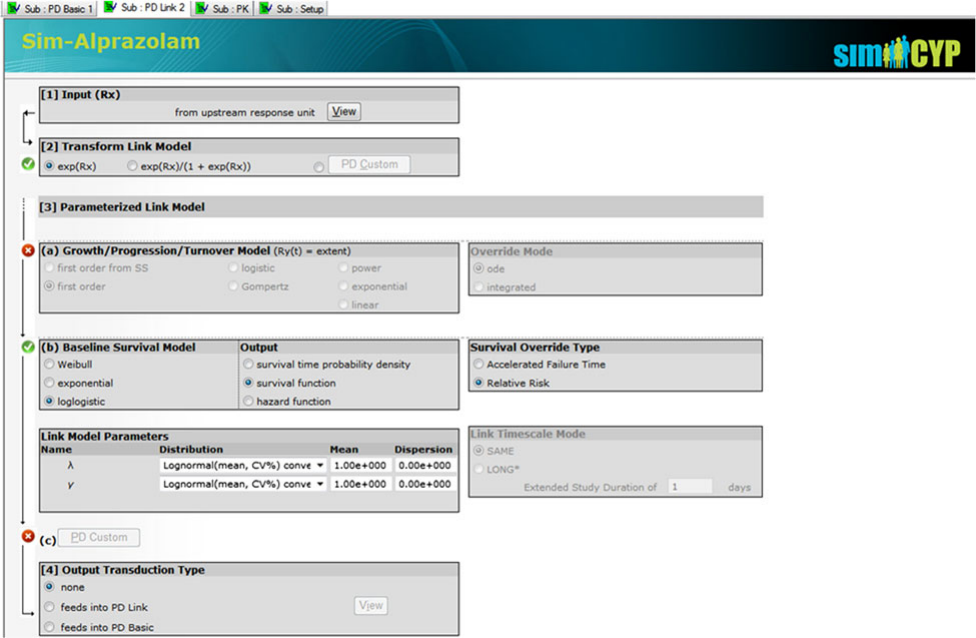
**Simcyp screen representing a PD link response unit: input from the previous unit can go through a Link transform model and then feed into either a growth/progression/turnover model, survival model or custom scripted model.**

Two types of PD Response Unit have so far been implemented: the PD Basic Unit (Version 11) and the PD Link Unit (Version 12). Just one PD Basic Unit is all that is needed to represent elementary PD (such as a Hill model or E_max_ response) and a PBPK compartment providing a concentration or amount driving the response can be selected from a simple dropdown list; thus a specialist in metabolism or biopharmaceutical science does not need further training to add a quick preview of possible PD effects, consequent to some simulated PK interaction or a formulation change. Nevertheless, with a PD Link Unit attached, Simcyp extends the offered response models to include many indirect models via link models well known to pharmacometricians and PK/PD modellers, combining concepts from Generalized Linear Models (McCullagh and Nelder 1989), Survival Analysis (Kalbfleisch and Prentice 2002), empirical disease progression (Chan and Holford 2001) and “Indirect Physiological PK/PD” (Dayneka et al. 1993). Figure 5 shows the screen representing the PD Link Unit.

PD Response Units are themselves subdivided into a sequence of data processing steps with associated model choices from unit input to unit output and this is represented on-screen as a flow from top to bottom. Moreover, a Simcyp response comprising just two connected PD Basic Units can be cast at different mechanistic levels according to a user-chosen transduction mode: other than just empirical mode; operational mode may be used to capture receptor binding-activation (upstream) and stimulus–response (downstream) with the efficient transducer-ratio parameterisation of operational agonism models (Black et al. 1985); or intrinsic efficacy mode representing intricacies more familiar to receptor pharmacologists (Stephenson 1956 Karlin 1967), with explicit receptor abundance (at least) as a system property to be pulled from a Simcyp population library. As of Simcyp Version 12, PK/ADME extensions, particularly for biologic drugs with high target affinity, have included target-mediated drug disposition (TMDD) models (Mager and Jusko 2001) where target receptor binding affects drug disposition profiles: from Version 12 Release 2 these models are also available for PD response.

Completion of the full DAG framework would require two further items, namely a PD Interaction Unit to join responses of different compounds according to phamacodynamic interaction models and a response splitting motif which allows unit outputs to input to more than one downstream unit.

The framework so far described, and included in Version 11, is sufficient to allow a user to input elementary descriptions of inter-individual variability in PD model parameter distribution as well as optimise the population mean parameters (possibly within the context of a high-dimensional PBPK model). However it is important ultimately to provide cross-links from potential predictors in Simcyp’s virtual populations (“covariates”) which condition PD model parameters to account for PK/PD correlation mechanisms to underpin pharmacometrics concepts active within the NONMEM community: the intrinsic efficacy and custom scripting facilities (see next section) already go some way towards this ideal framework.

In addition to simple time-independent and PK/PD profiles modes, the PD Simulator in Version 12 also accommodates possibilities of time-summaries converting profile responses into time-independent responses and the “LONG” timescale mode feature in a Link Response Unit which allows some link models (such as survival models) to be run on an extended timescale after a time-summarised or otherwise time-independent input.

### Custom PD scripting

A facility allowing user-scripted custom models to be inserted as replacement processing steps into the PD framework was developed in Version 12 Release 2. Lua **(2)** was selected for (first) implementation of this requirement as a freely available, lightweight and efficient embedded scripting language with widespread support. In particular, to function well, a script interpretation needs to be very fast. It is likely that later versions of Simcyp will interface more extensively with other (typically compiled) modelling languages familiar to pharmacometricians and PK/PD scientists (such as Pharsight Modelling Language (PML)). In any case, the provision of scripting extends the range of models that Simcyp can simulate.

The execution context of user-scripted code in a population Simulator with hierarchical variability and many predefined models needs careful consideration. In Simcyp Version 12 Release 2 this is primarily controlled by the positioning of a script as a replacement for a specific response step in a specific response unit for a particular compound. Simcyp also provides several placeholders in its code for users to implement call-back functions for different simulation contexts. Simcyp Version 12 Release 2 includes a dedicated Lua script editor (based on Scintilla editing software components **(3)**) which supplies templates for such code. It also implements a library of Simcyp functions callable from Lua scripts: for storing variables in the Simulator and accessing or manipulating elements of the PK and PD simulation. This notably includes read access to potential covariates in a virtual population.

The custom scripting module also facilitates handling of user defined differential equations. For this purpose, a block of state variables and corresponding time gradients are reserved for user scripting of ordinary differential equations (ODEs). Just as the built in engine has access to the state variables and predict time gradients for the generic ODE solvers, so will the custom defined differential equations do the same for the user block, and this block will be re-indexed into the full state array by Simcyp.

User step functions will be called many times as the ODE solver updates the model predictions for each time-step so script efficiency is crucial here. Benchmark performance tests of Lua prototype ODE code have however demonstrated impressive performance: these tests contributed to the selection of Lua over other possible scripting options.

### Expansion of differential equation solvers

PBPK models are dynamic models constructed using differential equations. Therefore, having a robust, efficient and reliable differential equation solver engine is an essential part of any software that deals with such models. As the number and complexity of implemented models within the Simulator are increasing, the possibility of encountering stiff ordinary differential equations (ODEs) has increased. Therefore it was decided to introduce a second differential equation solver that can efficiently handle stiff differential equations. In practice the stiffness of ODEs is a transient phenomenon: i.e. an ODE may be stiff in some interval and non-stiff in others. Hence, it is desirable to choose a scheme which can dynamically follow the qualitative behaviour of the ODE. After assessing a range of solvers it was decided to use LSODE (Livermore Solver for Ordinary Differential Equations) (Radhakrishnan and Hindmarsh 1993). LSODE is a very robust method that combines the capabilities of the GEAR approach (Gear 1971) and solves explicitly given stiff and non-stiff system of ODE. It offers a number of features (see (Hindmarsh 1983)) which are more convenient, flexible, portable, and easier to install in software libraries.

A variant of LSODE called LSODA (the suffix A stands for automatic) (Petzold 1983) was adopted. The algorithm used within LSODA has the capability to switch automatically between stiff and non-stiff methods. This is very convenient for the Simulator as it optimises the simulation speed. Moreover LSODA is known to be potentially more efficient than the basic LSODE when the nature of the problem changes between stiff and non-stiff in the course of the solution. The Livermore solver was introduced in Simcyp Version 9 in addition to the fifth-order Runge–Kutta method (Jamei et al. 2009a). The fifth-order Runge–Kutta method is very robust for the majority of simulations. Hence we kept both solvers and set the Runge–Kutta as the default solver. Users can switch between the solvers and there are cases where according to the selected combination of models the Simulator suggests or enforces selecting the Livermore solver.

Both of the solvers are coded in C++ and optimised to address specific needs in dealing with complex dose administration regimens. The ODE solvers are located in an independent module which is completely separated from the other modules so it can be used as an independent engine and called up wherever needed even outside the Simulator.

### Memory usage and speed maintenance

#### Memory management

Due to the number of additional outputs required in each version as well as the need for larger population sizes, improvements had to be done on the way the generated data are maintained. The first stage for improvements was the removal of duplicated data; this was mostly in the area of how individuals’ data were stored. Removing duplicated data significantly increased the possible number of simulated subjects and also improved the time taken to create the individuals data.

The next step was to improve the way concentration time profile data were handled. This was accomplished via two stages of improvements. Firstly the stored data were split so that systemic plasma concentrations were kept in greater detail than the rest of the concentration time profiles. Secondly a compression algorithm was introduced, with a pre-processing step that increases the compressibility. The pre-processing step takes advantage of the fact that both integer and floating point data use 4 bytes of memory and keeps the integer value of subtraction of two consecutive values rather than their absolute values. Without pre-processing representative data were compressed to 40% of original size; however after pre-processing they reduced to 25%.

### Using multiprocessor and multicore advantages

The advancement of laptops and workstation machines has meant that support for multiple processing cores is paramount for modern software. Since the main computational burden in the Simulator is solving simultaneous sets of equations, there were limitations on how to approach the use of these additional cores. Nevertheless, given the population based nature of the simulations one candidate solution was to simulate multiple individuals concurrently. Interestingly, there were limitations on the gained efficiency when the computation load was divided among different cores. Testing different computer configurations revealed that the main consideration for the scalability appears to be the size of the processor cache, the larger the cache the greater the scalability. Therefore, whenever multiprocessor and/or multicore machines are available the Simulator has been designed to take advantage of them.

### Maintaining the simulation speed

As the complexity of models and the number of differential equations increase so does the possibility of slowing down the simulation speed. To obtain quantitative measures of the speed performance of the Simulator an in-house package called Profiler is developed and used to measure the runtime duration for a wide range of internal tasks. The Profiler statistics are regularly assessed during the development process and in particular when new modules are introduced and compared against the historical records. Typical types of collected data include which functions in the code are being called, how many times, how much time is spent inside the function and in many cases how much time was spent on specific sections of code inside those functions. Whenever a speed reduction is observed the relevant functions are investigated and are possibly optimised to regain the lost speed. Sometimes the saving of only a few milliseconds in a function can add up to large savings over the course of an entire simulation.

One of the major tasks undertaken as part of these optimization processes was to rewrite portions of both of the Simcyp Simulators differential equation solvers using processor instruction sets which take advantage of Single Input Multiple Data (SIMD) facilities known as Streaming SIMD Extension (SSE) Intrinsics **(4)**. Machines supporting SSE2, which allows multiple floating point instructions to be carried out simultaneously, have been available since 2001 (Intel, Santa Clara, CA, USA) and 2003 (AMD, Sunnyvale, CA, USA) and are now included in all commercially available x86 processors. The differential equation solvers in the Simcyp Simulator use double precision data, so application of SSE2 instructions allowed two calculations to be performed for each processor clock cycle thus improving the speed and efficiency. In order to make full use of the power of these instructions the data has to be specifically aligned to 16 byte boundaries; also the size of the data must always be an even number, so if an odd number of items is required a dummy set must be added to the end. The changes significantly improved the overall simulation time of complex models by approximately 10% for the Runge Kutta solver and 20% for the Livermore solver.

## Conclusions

The iterative and incremental development of the Simcyp platform has facilitated its rapid expansion and the consortium guidance has kept it in line with users’ requirements. Applying the concept of separation of databases and models has assisted with implementing the systems pharmacology paradigm. Further, since scientists are directly involved in the design and development process and working closely with developers the whole software development life cycle has improved and shortened. Implementing the auto and regression testing processes has reduced the burden of testing and the SDM systems has smoothened the integration and tracking of data in the databases. As the role and applications of model-based drug development approach increase so does the need for peripheral modules (e.g. PD, PE, ASA, Batch processor, etc.) that can seamlessly connect and interact with variety of different models and databases. Although more than a decade of internal experience and many more years of external knowledge have gone into the development of the Simulator there is a long way to go and lots more development and expansions are yet to come. Handling of large molecules with full PBPK models, expansion of the current models to all compound types and expansion of the population databases to other special groups (e.g. geriatric populations, different ethnicities) are only some of the potential future developments. More exciting and challenging directions include identifying and incorporating response covariates, incorporating physiological changes in disease progression and addressing safety issues such as cardiotoxicity, nephrotoxicity, hepatotoxicity and neurotoxicity with mechanistic models. It is also envisaged to connect the Simulator to other packages such as Tripos (a Certara company, St. Louis, USA) and Pharsight (a Certara company, St. Louis, USA) products to apply the MBDD paradigm in different phases of the drug discovery and development cycle.

## Websites

**(1)** The R-Project for Statistical Computing [http://www.r-project.org]

**(2)** Scintilla and SciTE [http://www.lua.org]

**(3)** The Programming Language Lua [http://www.scintilla.org]

**(4)** Microsoft Developer Network - Visual Studio .Net 2003 MMX, SSE, and SSE2 Intrinsics 05/03/2013 [http://msdn.microsoft.com/en-us/library/y0dh78ez(v=vs.71).aspx]

## Abbreviations

ADME: Absorption distribution, metabolism, and excretion
PK: Pharmacokinetics
PD: Pharmacodynamics
PBPK: Physiologically-based pharmacokinetic
MBDD: Model based drug development
DDI: Drug-drug interaction
SDM: Simcyp data management
TFS: Team foundation server
PML: Pharsight modelling language
NONMEM: Nonlinear mixed effects modelling
NLME: Nonlinear mixed effects
GLM: Generalised linear model
COM: Component object model
ODE: Ordinary differential equation
LSODE: Livermore solver for ordinary differential equations
LSODA: LSODE with automatic switching
SSE: Streaming SIMD extensions
SIMD: Single instruction multiple data
NCC: National computing centre
IT: Information technology
XML: Extensible markup language
ASA: Automated sensitivity analysis
PE: Parameter estimation
ADAM: Advanced dissolution absorption and metabolism.

## References

[CR1] Black JW, Leff P, Shankley NP, Wood J (1985). An operational model of pharmacological agonism: the effect of E/[A] curve shape on agonist dissociation constant estimation. Br J Pharmacol.

[CR2] Bouzom F, Ball K, Perdaems N, Walther B (2012). Physiologically based pharmacokinetic (PBPK) modelling tools: how to fit with our needs?. Biopharm Drug Dispos.

[CR3] Chan PL, Holford NH (2001). Drug treatment effects on disease progression. Annu Rev Pharmacol Toxicol.

[CR4] Dayneka NL, Garg V, Jusko WJ (1993). Comparison of four basic models of indirect pharmacodynamic responses. J Pharmacokinet Biopharm.

[CR5] Dempster AP, Laird N, Rubin DB (1977). Maximum likelihood from incomplete data via the EM algorithm. J R Stat Soc Series B.

[CR6] Gear CW (1971). Numerical initial value problems in ordinary differential equations.

[CR7] Goldberg DE (1989). Genetic algorithms in search, optimization, and machine learning.

[CR8] Guckenheimer S, Loje N (2012). Visual studio team foundation server 2012: adopting agile software practices, from backlog to continuous feedback (Microsoft windows development).

[CR9] Hindmarsh AC (1983). ODEPACK, a systematized collection of ODE solvers. IMACS transactions on scientific computation, 10th IMACS world congress on systems simulation and scientific computation, August 8–13, 1982.

[CR10] Hooke R, Jeeves TA (1961). Direct search solution of numerical and statistical problems. J Assoc Comp Mach.

[CR11] Jamei M, Marciniak S, Feng K, Barnett A, Tucker G, Rostami-Hodjegan A (2009). The Simcyp^®^ population-based ADME simulator. Expert Opin Drug Metab Toxicol.

[CR12] Jamei M, Dickinson GL, Rostami-Hodjegan A (2009). A framework for assessing inter-individual variability in pharmacokinetics using virtual human populations and integrating general knowledge of physical chemistry, biology, anatomy, physiology and genetics: a tale of ‘bottom-up’ vs ‘top-down’ recognition of covariates. Drug Metab Pharmacokinet.

[CR13] Kalbfleisch JD, Prentice RL (2002). The statistical analysis of failure time data.

[CR14] Karlin A (1967). On the application of “a plausible model” of allosteric proteins to the receptor for acetylcholine. J Theor Biol.

[CR15] Larman C, Basili VR (2003). Iterative and incremental development: a brief history. Computer.

[CR16] Mager DE, Jusko WJ (2001). General pharmacokinetic model for drugs exhibiting target-mediated drug disposition. J Pharmacokinet Pharmacodyn.

[CR17] Martin RC (2011). Agile software development, principles, patterns, and practices.

[CR18] McCullagh P, Nelder JA (1989). Generalized linear models.

[CR19] Nelder JA, Mead R (1965). A simplex method for function minimization. Comput J.

[CR20] Petzold L (1983). Automatic selection of methods for solving stiff and nonstiff systems of ordinary differential equations. SIAM J SCI Stat Comput.

[CR21] Radhakrishnan K, Hindmarsh AC (1993). Description and Use of LSODE, the Livermore solver for ordinary differential equations. Lawrence Livermore national laboratory, university of California.

[CR22] Rostami-Hodjegan A (2012). Physiologically based pharmacokinetics joined with *in vitro*-*in vivo* extrapolation of ADME: a marriage under the arch of systems pharmacology. Clin Pharmacol Ther.

[CR23] Rowland M, Peck C, Tucker G (2011). Physiologically-based pharmacokinetics in drug development and regulatory science. Ann Rev Pharmacol Toxicol.

[CR24] Sims C, Johnson HL (2012). Scrum: A breathtakingly brief and agile introduction.

[CR25] Stephenson RP (1956). A modification of receptor theory. Br J Pharmacol Chemother.

[CR26] Teorell T (1937). Studies on the diffusion effect upon ionic distribution: II. Experiments on ionic accumulation. J Gen Physiol.

[CR27] Zhao P, Zhang L, Grillo JA, Liu Q, Bullock JM, Moon YJ, Song P, Brar SS, Madabushi R, Wu TC, Booth BP, Rahman NA, Reynolds KS, Berglund EG, Lesko LJ, Huang SM (2011). Applications of physiologically based pharmacokinetic (PBPK) modeling and simulation during regulatory review. Clin Pharmacol Ther.

[CR28] Zhao P, Vieira ML, Grillo JA, Song P, Wu TC, Zheng JH, Arya V, Berglund EG, Atkinson AJJ, Sugiyama Y, Pang KS, Reynolds KS, Abernethy DR, Zhang L, Lesko LJ, Huang SM (2012). Evaluation of exposure change of nonrenally eliminated drugs in patients with chronic kidney disease using physiologically based pharmacokinetic modeling and simulation. J Clin Pharmacol.

